# The Causal Relationship Between Asthma and Hippocampal Volume: A Study Based on Bidirectional Mendelian Randomization Analysis

**DOI:** 10.1002/brb3.70560

**Published:** 2025-05-26

**Authors:** Dandan Han, Shaohua Zhao

**Affiliations:** ^1^ Medical Imaging Center Xuchang Central Hospital Affiliated to Henan University of Science and Technology Xuchang P. R. China; ^2^ Department of Plastic Surgery Xuchang Central Hospital Affiliated to Henan University of Science and Technology Xuchang P. R. China

**Keywords:** asthma, brain imaging‐derived phenotypes, genome‐wide association study, hippocampal volume, Mendelian randomization

## Abstract

**Background:**

Asthma is a common chronic respiratory disease, and its potential association with the central nervous system has garnered increasing attention in recent years. While observational studies suggest that asthma may affect hippocampal structure and function through mechanisms such as chronic inflammation and hypoxia, its causal relationship remains unclear.

**Methods:**

In this study, we employed a two‐sample Mendelian randomization (MR) analysis, utilizing large‐scale genome‐wide association study (GWAS) data to systematically investigate the potential causal relationship between asthma and hippocampal volume. Data from a GWAS of asthma involving 155,386 individuals of European ancestry and GWAS imaging‐derived phenotypes (IDPs) of hippocampal volume from 33,219 European individuals were analyzed.

**Results:**

The results revealed a significant negative correlation between asthma and multiple hippocampal IDPs (*P*_FDR_ < 0.05), indicating that asthma may contribute to reduced hippocampal volume. We identified 19 independent SNPs significantly associated with asthma (*P* < 5×10⁻⁸), of which 16 SNPs were retained after clumping (r^2^ < 0.001) and harmonization. Sensitivity analyses revealed no heterogeneity or horizontal pleiotropy, and reverse MR analysis did not support a causal effect of hippocampal volume on asthma.

**Conclusion:**

Our study provides genetic evidence for a causal relationship between asthma and changes in hippocampal volume, highlighting the need for closer monitoring and intervention in the neurocognitive health of asthma patients in clinical practice. Future studies should explore the causal relationship between asthma and brain structural changes across different racial groups and asthma subtypes, as well as the underlying biological mechanisms.

## Graphical abstract

This study reveals a causal relationship between asthma and reduced hippocampal volume using Mendelian randomization analysis. Asthma may negatively impact hippocampal structure via chronic inflammation, hypoxia, and long‐term glucocorticoid use, suggesting the need for monitoring neurocognitive health in asthma patients.

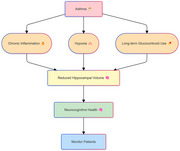

## 1 Introduction

Asthma is a prevalent chronic respiratory disorder characterized by chronic airway inflammation, hyperreactivity, and reversible airflow limitation (Russell et al. 2024; Savin et al. 2023). Recently, growing attention has been directed toward its potential link with the central nervous system (Han et al. 2022; Kroll and Ritz 2023). Asthma patients frequently present with various neuropsychiatric comorbidities, including anxiety, depression, and cognitive impairments, suggesting that asthma may influence brain structure and function through complex mechanisms (Caulfield 2021; Rhyou and Nam 2021). The hippocampus, a critical brain region involved in emotion regulation, memory, and learning, is often implicated in neuropsychiatric disorders such as depression and Alzheimer's disease, both of which are characterized by alterations in hippocampal volume (Langella et al. 2024; Paolini et al. 2023). Additionally, the hippocampus is highly susceptible to hypoxia and inflammation, which are commonly induced by chronic asthma (Churilova et al. 2022; Shaker et al. 2021). Systemic inflammation, intermittent hypoxia, and psychological stress associated with chronic asthma may negatively affect hippocampal structure. Previous studies have reported that cognitive decline and emotional disorders in asthma patients may be linked to hippocampal volume reduction (Esmaeilpour et al. 2024; Kroll et al. 2018; Ritz et al. 2020). Therefore, investigating the potential causal relationship between asthma and hippocampal volume is of significant clinical and scientific interest.

While existing research has suggested an association between asthma and structural abnormalities in the hippocampus, most of these studies are observational in nature, limiting the ability to determine causality (Carlson et al. 2017; Ritz et al. 2020). Observational studies are often confounded by various factors and cannot fully exclude the possibility of reverse causation. Mendelian randomization (MR), a method of causal inference, uses genetic variants as instrumental variables (IVs) to minimize confounding bias, offering a more robust approach to determining causal relationships (Burgess, Small, and Thompson 2017; Emdin et al. 2017; Swanson et al. 2017). However, no prior studies have explicitly examined the causal relationship between asthma and changes in hippocampal volume.

This study addresses this gap by utilizing large‐scale genome‐wide association study (GWAS) data and applying a two‐sample MR framework to systematically assess the potential causal effect of asthma on hippocampal volume. The aim is to provide robust causal evidence for the effects of asthma on hippocampal structural changes, contributing to a better understanding of the central nervous system complications of asthma.

## 2 Methods

### 2.1 Study Design

**FIGURE 1:**
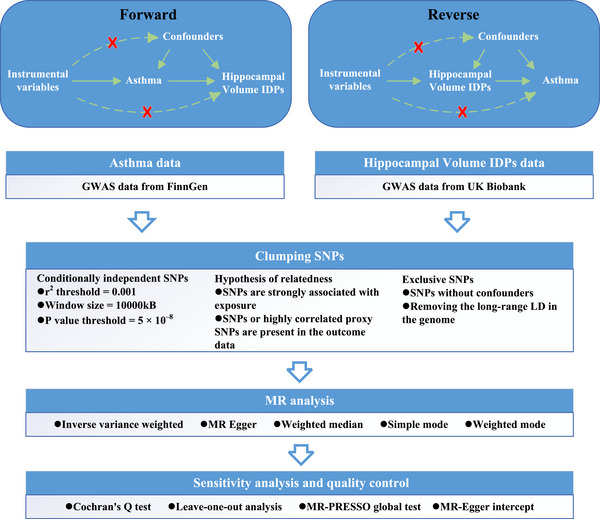
Workflow for causal inference between asthma and hippocampal volume IDPs.

The design of this study followed a standard two‐sample MR framework (Figure 1). Three core assumptions underpin MR analysis: (1) the genetic variant must be strongly associated with the exposure (relevance); (2) the genetic variant must be independent of any confounders (independence); and (3) the genetic variant must influence the outcome only through the exposure and not through any alternative pathways (exclusivity). Under these assumptions, single nucleotide polymorphisms (SNPs) were selected as IVs for two‐sample MR analysis (Emdin et al. 2017).

### 2.2 Data Sources

In the forward MR analysis, asthma was considered the exposure, and hippocampal volume imaging‐derived phenotypes (IDPs) were the outcome variables. The GWAS summary statistics for asthma were derived from the FinnGen project, comprising 155,386 European individuals (19,573 cases and 135,813 controls) with coverage of 9,851,867 SNPs. These data are accessible through the MRC IEU OpenGWAS project database (https://gwas.mrcieu.ac.uk/). The GWAS data for hippocampal volume IDPs were obtained from eight datasets, which included structural MRI‐derived measurements of left and right hippocampal volumes (mm^3^), hippocampal gray matter volumes (mm^3^), and whole‐hippocampus subfield volumes generated by automated segmentation tools (e.g., FMRIB's FIRST and FAST algorithms) (Armstrong et al. 2020; S. M. Smith et al. 2021). The sample sizes ranged from 31,968 to 33,219 European individuals. These IDPs were derived from high‐resolution T1‐weighted MRI scans acquired using standardized Siemens Skyra 3T systems with consistent imaging protocols (voxel resolution: 1×1×1 mm; field‐of‐view: 208×256×256 mm). For example, “T1 image left hippocampus volume” represents the total volume of the left hippocampus segmented from T1‐weighted images, while “right hippocampus grey matter volume” quantifies the volume of gray matter tissue within the right hippocampus. Full definitions of all IDPs, including acquisition parameters and segmentation methods, are summarized in Supplementary Table 1. Data summaries are available in Supplementary Tables 2 and 3.

Since this study utilized publicly available GWAS summary statistics, no additional ethical approval was required.

### 2.3 Instrumental Variable Selection

We selected SNPs as IVs based on three core criteria. First, SNPs were required to exhibit genome‐wide significant associations with asthma (*P* < 5×10⁻⁸) in the exposure GWAS. Second, to ensure independence, SNPs were clumped for linkage disequilibrium using stringent thresholds (r^2^ < 0.001 within a 10,000 kb window), retaining only the most strongly associated SNP per locus. Third, SNPs associated with hippocampal pathology or its risk factors (e.g., Alzheimer's disease, Parkinson's disease, depression, or traumatic brain injury) were excluded by cross‐referencing the LDlink database (https://ldlink.nih.gov/) and applying a genome‐wide significance threshold (*P* < 5×10⁻⁸) (Babcock et al. 2021; Idunkova et al. 2023; Le Maître et al. 2018; Terreros‐Roncal et al. 2021; Weston et al. 2021). Additionally, SNPs with mismatched alleles between exposure and outcome datasets or ambiguous palindromic SNPs (e.g., A/T or C/G alleles with intermediate allele frequency >0.42) were removed during harmonization. Finally, IV strength was validated using the F‐statistic (F = β^2^ / SE^2^), with F > 10 for all SNPs, ensuring robustness against weak instrument bias (Burgess and Thompson 2011).

### 2.4 MR and Sensitivity Analyses

Inverse variance weighting (IVW) was used as the primary analysis method, which provides the most precise causal estimate when all SNPs are valid instruments (Burgess, Bowden, et al. 2017). Supplementary analyses included MR‐Egger, weighted median, simple mode, and weighted mode methods. MR‐Egger provides robust causal estimates when IVs are invalid, while the weighted median method offers reliable estimates when more than half of the SNPs are valid (Bowden et al. 2015, 2016). Simple mode and weighted mode analyses were used to further confirm the robustness of the results.

Heterogeneity was assessed using Cochran's Q statistic, and a *P*‐value < 0.05 indicated the presence of heterogeneity, prompting the use of random‐effects MR models (Greco M et al. 2015). The MR‐Egger intercept test was used to evaluate horizontal pleiotropy, with a *P*‐value < 0.05 suggesting pleiotropy that could influence the reliability of the results. Outliers were identified using the MR‐PRESSO global test, and leave‐one‐out analysis was employed to assess the impact of individual SNPs on the findings (Hartwig et al. 2017).

### 2.5 Data Processing and Reporting

All analyses were conducted using the Two‐Sample MR and MRPRESSO packages in R version 4.4.0. The results were reported as odds ratios (OR) with 95% confidence intervals (CI). False discovery rate (FDR) correction was applied for multiple testing, with *P*_FDR_ < 0.05 considered statistically significant. The results were presented in tables and figures to ensure transparency and clarity.

Additionally, we conducted a reverse MR analysis to explore the potential causal relationship between hippocampal volume IDPs and asthma, using the same settings and methods as the forward MR analysis.

## 3 Results

### 3.1 Forward MR Analysis of Instrumental Variables

From the GWAS data for asthma, we identified 19 SNPs that were significantly associated and independent, with F‐statistics ranging from 29.737 to 95.760, ruling out weak instrument bias (Supplementary Table 4). After further refinement, 16 SNPs were included in the MR analysis (Table 1).

**TABLE 1:** Instrumental variables used for Mendelian randomization analysis.

Number	SNP	Chr	Position	Effect allele	Beta	SE	*P*‐value	*F*‐statistic
1	rs11678975	2	103043739	A	0.095	0.013	3.00E‐13	53.515
2	rs62192043	2	242711282	A	−0.107	0.014	4.63E‐14	56.992
3	rs72837868	2	112010094	G	0.120	0.019	7.21E‐11	42.355
4	rs1837253	5	110401872	C	0.137	0.014	1.15E‐22	95.760
5	rs6894249	5	131797547	G	0.091	0.012	1.07E‐14	59.866
6	rs1010473	6	90856878	T	−0.081	0.014	2.87E‐09	35.129
7	rs35242582	6	32600057	G	−0.129	0.015	8.59E‐19	78.553
8	rs4713555	6	32575524	T	−0.079	0.013	3.71E‐09	34.581
9	rs7035413	9	6243119	G	0.131	0.015	3.42E‐18	75.725
10	rs827631	10	9015230	A	0.095	0.015	2.44E‐10	40.027
11	rs7126418	11	76292573	T	0.071	0.012	3.50E‐09	34.810
12	rs9517711	13	100074280	A	0.066	0.012	2.49E‐08	30.854
13	rs17293632	15	67442596	T	0.098	0.013	2.02E‐13	53.705
14	rs74630264	16	27316975	A	−0.159	0.022	3.46E‐13	53.063
15	rs8074437	17	38076137	G	0.101	0.012	2.86E‐17	71.324
16	rs72699	17	47328890	C	−0.065	0.012	4.65E‐08	29.971

Abbreviations: Chr: chromosome; SE: standard error; SNP: single nucleotide polymorphism.

### 3.2 MR and Sensitivity Analyses

**FIGURE 2:**
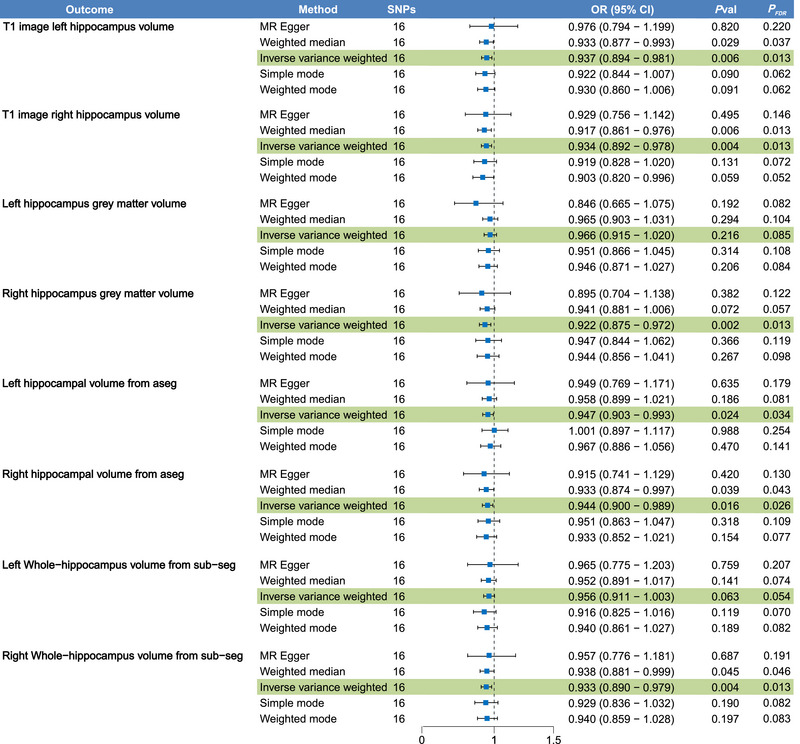
Comprehensive results of forward Mendelian randomization analysis.

**FIGURE 3:**
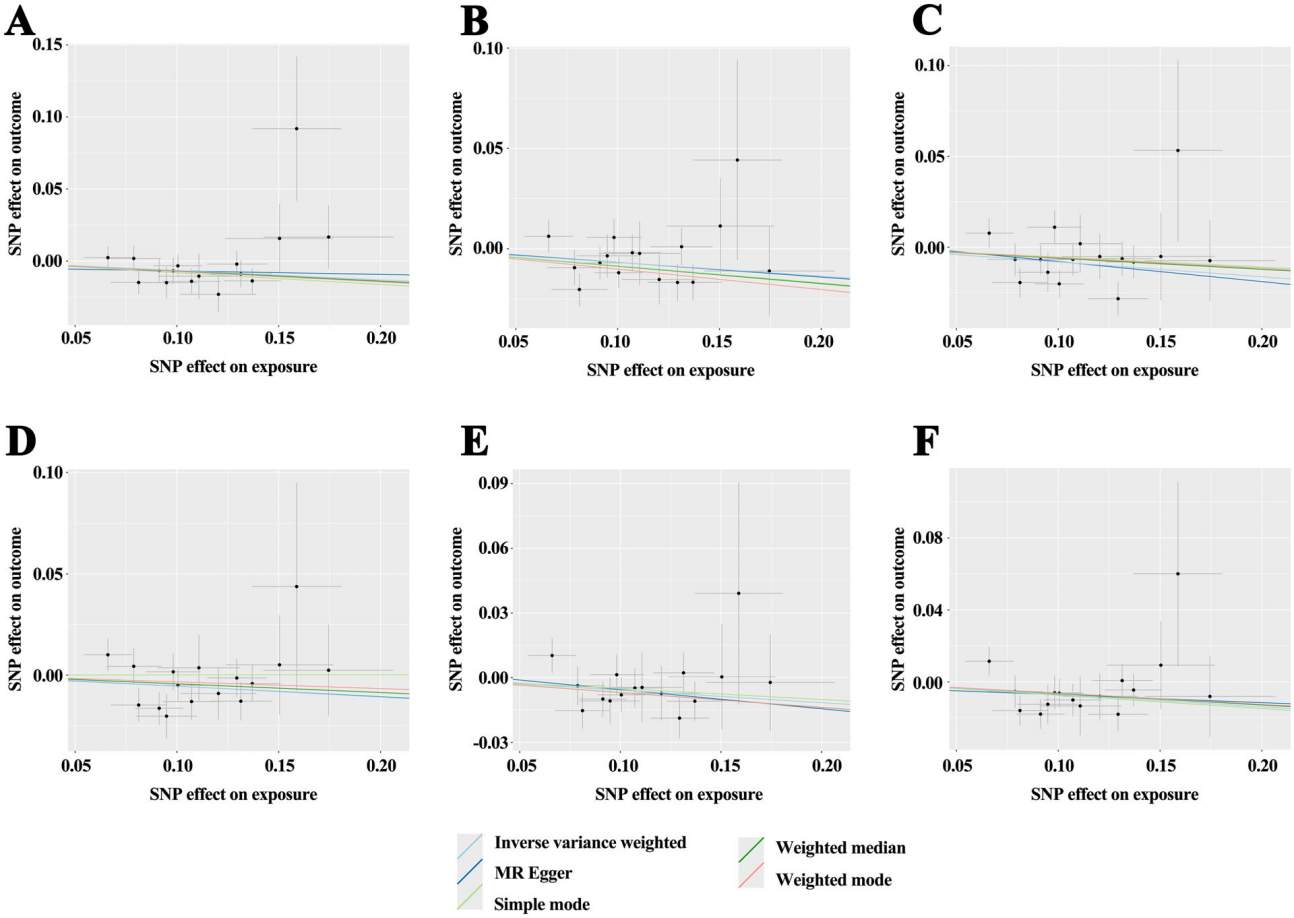
Scatter plots illustrating the MR results of asthma on hippocampal volume: (A) T1 image left hippocampus volume, (B) T1 image right hippocampus volume, (C) right hippocampus grey matter volume, (D) left hippocampal volume from aseg, (E) right hippocampal volume from aseg, and (F) right whole‐hippocampus volume from sub‐seg.

As shown in Figure 2 and Supplementary Table 5, the forward MR analysis using the IVW method indicated a negative causal relationship between genetically predicted asthma and several hippocampal volume IDPs, including T1 image left hippocampus volume (OR = 0.937, 95% CI = 0.894–0.981, *P*_FDR_ = 0.013), T1 image right hippocampus volume (OR = 0.934, 95% CI = 0.892–0.978, *P*_FDR_ = 0.013), right hippocampus gray matter volume (OR = 0.922, 95% CI = 0.875–0.972, *P*_FDR_ = 0.013), left hippocampal volume from aseg (OR = 0.947, 95% CI = 0.903–0.993, *P*_FDR_ = 0.034), right hippocampal volume from aseg (OR = 0.944, 95% CI = 0.900–0.989, *P*_FDR_ = 0.026), and right whole‐hippocampus volume from sub‐seg (OR = 0.933, 95% CI = 0.890–0.979, *P*_FDR_ = 0.013). However, the associations between asthma and left hippocampus gray matter volume (OR = 0.966, 95% CI = 0.915–1.020, *P*_FDR_ = 0.085) or left whole‐hippocampus volume from sub‐seg (OR = 0.956, 95% CI = 0.911–1.003, *P*_FDR_ = 0.054) did not reach statistical significance. The MR‐Egger, weighted median, simple mode, and weighted mode methods produced similar causal estimates (Figure 3).

**FIGURE 4:**
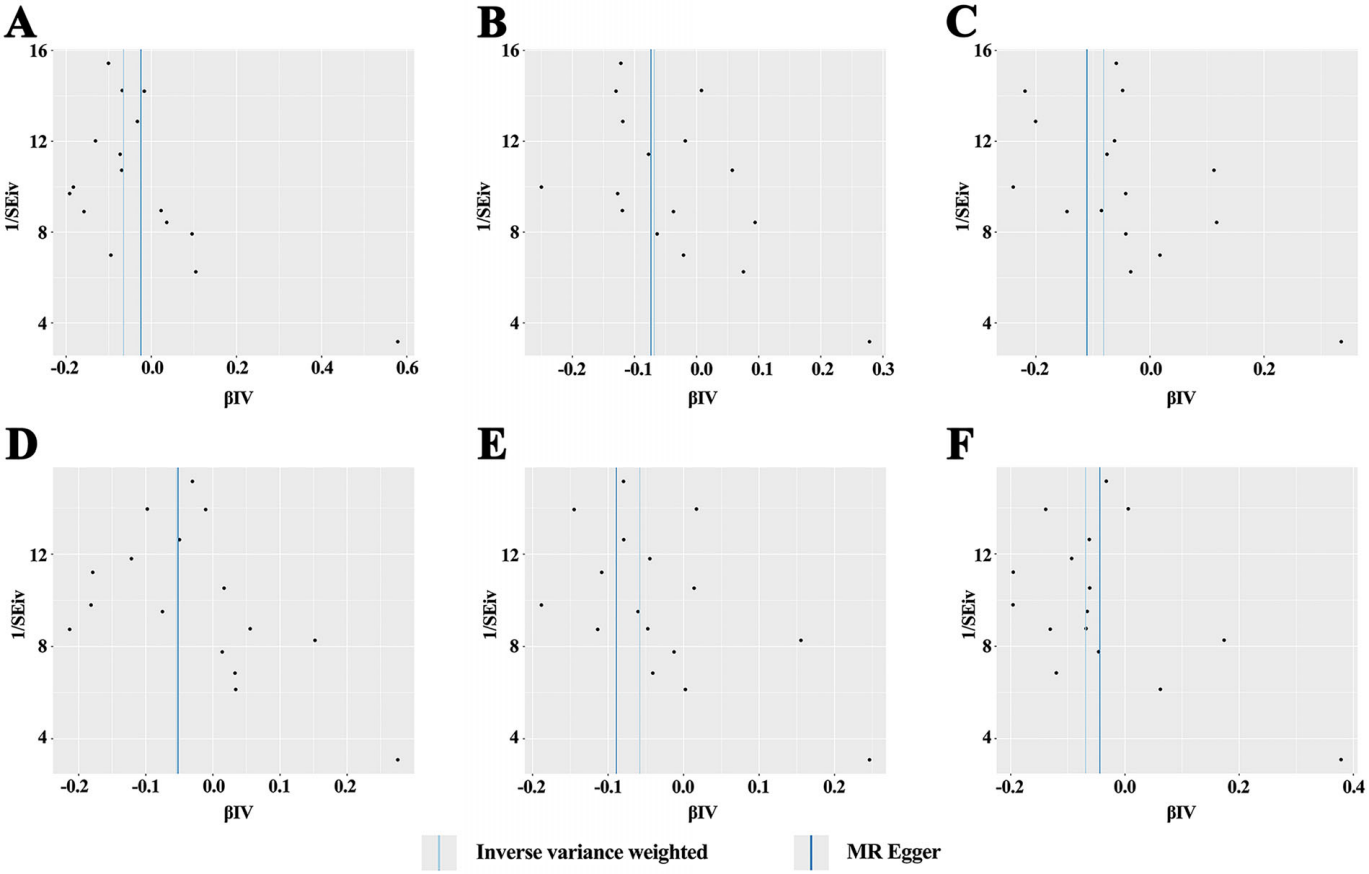
Funnel plots illustrating the MR results of asthma on hippocampal volume: (A) T1 image left hippocampus volume, (B) T1 image right hippocampus volume, (C) right hippocampus grey matter volume, (D) left hippocampal volume from aseg, (E) right hippocampal volume from aseg, and (F) right whole‐hippocampus volume from sub‐seg.

**FIGURE 5:**
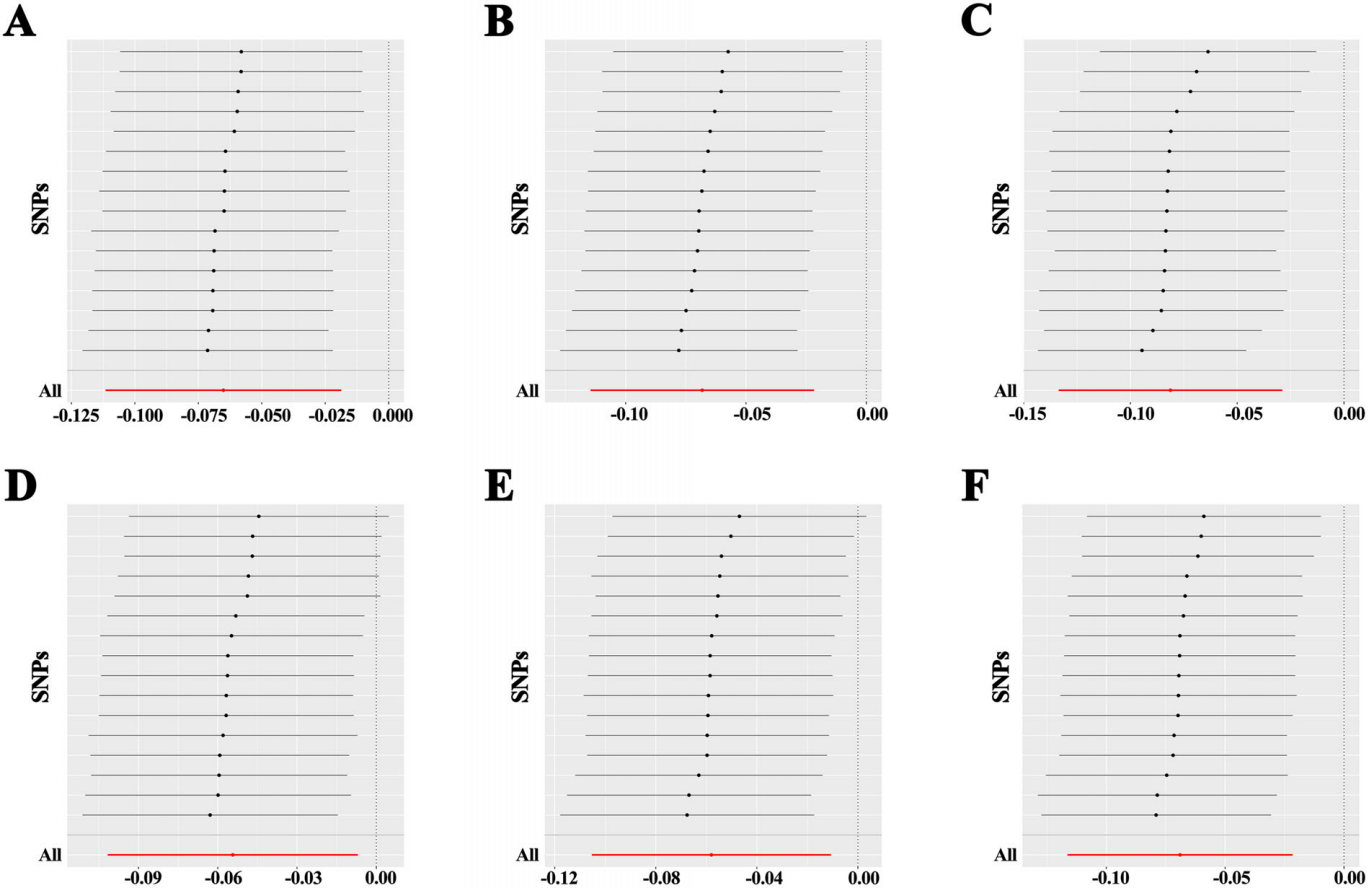
Leave‐one‐out sensitivity analysis for the impact of asthma on hippocampal volume: (A) T1 image left hippocampus volume, (B) T1 image right hippocampus volume, (C) right hippocampus grey matter volume, (D) left hippocampal volume from aseg, (E) right hippocampal volume from aseg, and (F) right whole‐hippocampus volume from sub‐seg.

Sensitivity analyses confirmed the robustness of the causal effect of asthma on hippocampal IDPs, with no evidence of heterogeneity in the IVW analysis (*P* > 0.05) (Supplementary Table 6). Funnel plots were also generated to visualize heterogeneity (Figure 4). The MR‐Egger intercept test did not detect horizontal pleiotropy (*P* > 0.05), and the MR‐PRESSO global test found no outliers (Supplementary Table 6). Leave‐one‐out analysis showed that the MR results were not driven by any single SNP (Figure 5). A forest plot of each SNP's causal effect on hippocampal IDPs is provided in Supplementary Figure 1. Overall, the sensitivity analyses confirmed the robustness of the MR findings.

### 3.3 Reverse MR Analysis

In the reverse two‐sample MR analysis, we used 6 to 14 SNPs for each hippocampal IDP as IVs to assess the causal effect of hippocampal volume on asthma. The F‐statistics ranged from 29.904 to 184.516, all exceeding the threshold of 10 (Supplementary Tables 7 and 8). Reverse MR analysis found no evidence of a causal effect of hippocampal volume IDPs on asthma risk (Supplementary Figure 2).

## 4 Discussion

This study used a two‐sample MR approach to investigate the potential causal relationship between asthma and hippocampal volume. The forward MR analysis showed a significant negative correlation between asthma and several hippocampal IDPs, including T1 image left hippocampus volume, T1 image right hippocampus volume, right hippocampus gray matter volume, left hippocampal volume from aseg, right hippocampal volume from aseg, and right whole‐hippocampus volume from sub‐seg. These results suggest that asthma may contribute to hippocampal volume reduction, and these findings were corroborated by various analytical methods, including MR‐Egger, weighted median, and mode‐based analyses. Sensitivity analyses revealed no evidence of heterogeneity or pleiotropy, supporting the robustness of the results. The reverse MR analysis did not support a causal effect of hippocampal volume on asthma, indicating that the causal relationship between asthma and hippocampal changes is likely unidirectional.

Asthma‐induced changes in hippocampal structure or function may involve several potential biological mechanisms, although the exact pathways remain unclear. Previous observational studies have reported reduced hippocampal volume in asthma patients, but these findings were limited by residual confounding (e.g., medication use, disease severity) and inability to infer causality (Carlson et al. 2017; Ritz et al. 2020). Our MR findings extend these observations by providing robust genetic evidence for a causal role of asthma in hippocampal atrophy, independent of traditional confounders. First, asthma is associated with chronic systemic inflammation, which may lead to central nervous system inflammation through the penetration of peripheral inflammatory factors across the blood‐brain barrier (Antunes et al. 2022; Kabata and Artis 2019; Ritz et al. 2019). In asthma patients, elevated levels of inflammatory factors such as IL‐1β, IL‐6, and TNF‐α can activate microglia, induce oxidative stress, and exert neurotoxic effects on hippocampal neurons, leading to hippocampal volume reduction and functional impairment (Liu et al. 2019). Additionally, the chronic hypoxic state induced by airway obstruction during asthma exacerbations may cause neuronal degeneration and apoptosis, further damaging hippocampal neurons and exacerbating inflammation, thus impairing hippocampal structure and function (Barhwal et al. 2020; Zhuang et al. 2018).

**FIGURE 6:**
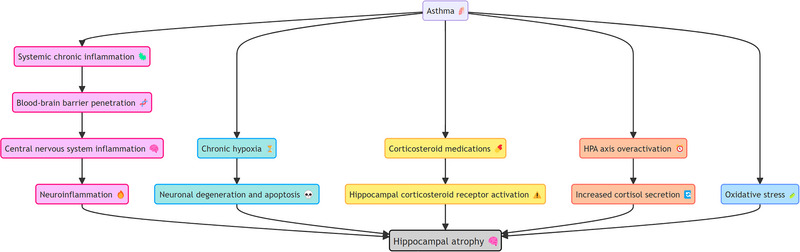
The potential biological mechanisms involved in asthma‐induced hippocampal atrophy.

The use of systemic glucocorticoids (e.g., oral prednisone or intravenous methylprednisolone), typically prescribed for severe asthma exacerbations or refractory cases, has been associated with hippocampal atrophy through chronic activation of glucocorticoid receptors (Brown et al. 2008; Nasereddin et al. 2024). Glucocorticoid receptors are highly expressed in the hippocampus, and chronic receptor activation may lead to neurodegenerative changes, impair synaptic function, and reduce neural plasticity, contributing to hippocampal volume reduction and cognitive decline (Kraus et al. 2022). Oxidative stress, which is prevalent in asthma patients due to the accumulation of free radicals, may also exacerbate hippocampal structural deterioration (Bejeshk et al. 2023). The hippocampus, with its high metabolic rate and lipid content, is particularly vulnerable to oxidative stress, a mechanism that may play a role in asthma‐related hippocampal damage (Abbah et al. 2022). Lastly, dysregulation of the neuroendocrine system, particularly characterized by excessive suppression of the hypothalamic‐pituitary‐adrenal (HPA) axis and elevated exogenous cortisol levels, has also been implicated in hippocampal atrophy (Brown et al. 2008; Conrad and Bimonte‐Nelson 2010; R. W. Smith et al. 2012). Prolonged elevated cortisol levels can induce neurodegenerative changes in the hippocampus, further impairing cognitive function. Overall, the changes in hippocampal structure and function induced by asthma may result from a combination of chronic inflammation, hypoxia, glucocorticoid use, oxidative stress, and neuroendocrine imbalances (Figure 6). However, further well‐designed studies are needed to clarify these mechanisms and confirm the causal relationship between asthma and hippocampal alterations.

The reverse MR analysis did not support a causal effect of hippocampal volume IDPs on asthma, suggesting that changes in hippocampal volume are likely a consequence of asthma rather than a cause. Asthma is a complex chronic disease with primary pathological mechanisms centered on airway inflammation and airflow limitation. While asthma may influence the central nervous system, there is no evidence to suggest that hippocampal volume changes would directly trigger asthma. However, emerging evidence highlights indirect neuroimmune pathways: the hippocampus interacts with the hypothalamus and amygdala to regulate sympathetic nervous system activity, which may modulate systemic inflammation and airway hyperreactivity via β2‐adrenergic receptor signaling (Chhatar and Lal 2021; Mueller et al. 2022). For instance, sympathetic nervous system activation can amplify pro‐inflammatory cytokine release (e.g., IL‐6) and mast cell degranulation, potentially exacerbating asthma pathophysiology (Pavón‐Romero et al. 2021). Thus, hippocampal dysfunction might indirectly influence asthma severity through neuroendocrine‐immune crosstalk, though this hypothesis requires further validation. Importantly, our MR findings do not support reverse causality, as hippocampal volume changes are more likely to reflect localized neurocognitive effects rather than drive asthma progression.

By utilizing the MR method, this study overcomes limitations in causal inference associated with observational studies. The use of genetic variants from GWAS as IVs in the MR approach effectively minimizes confounding factors and reverses causation, thereby enhancing the robustness of causal inference. The findings suggest that asthma may lead to hippocampal volume reduction, highlighting the potential impact of asthma on the central nervous system. Chronic inflammation, hypoxia, and long‐term use of glucocorticoids may be key factors contributing to hippocampal atrophy, which in turn may impair cognitive abilities and emotional regulation in asthma patients. Therefore, early identification and intervention for neuropsychiatric comorbidities in asthma, particularly in patients undergoing prolonged glucocorticoid therapy, may have important implications for protecting hippocampal function. Furthermore, this study employed multiple sensitivity analyses, including MR‐Egger, weighted median, and MR‐PRESSO, to confirm the robustness of the results, thereby strengthening the reliability of the study's conclusions. This study not only provides more robust evidence for the causal relationship between asthma and hippocampal volume but also offers new clinical perspectives on the neuropsychiatric monitoring and intervention of asthma patients.

Despite the strong evidence provided by this study, several limitations remain. First, the study data were primarily based on European populations, limiting the generalizability of the findings to other ethnic groups. Differences in standardization across data sources may also introduce measurement bias. Second, the heterogeneity of asthma (e.g., allergic versus non‐allergic subtypes) was not thoroughly addressed, potentially overlooking subtype‐specific effects on hippocampal volume (Xu et al. 2023). Additionally, the lack of granular data on asthma severity, disease duration, and medication use (e.g., systemic corticosteroids) limits our ability to disentangle their potential confounding effects on hippocampal structure. In summary, future studies addressing ethnic diversity, asthma heterogeneity, clinical severity, and long‐term neuroimaging trajectories are needed to validate and expand these findings.

## 5 Conclusion

This study is the first to reveal a potential causal relationship between asthma and changes in hippocampal volume through two‐sample MR analysis, suggesting that asthma patients may be at higher risk of cognitive impairment and emotional dysregulation. Asthma, as a chronic inflammatory disease, may affect hippocampal structure through chronic inflammation, hypoxia, long‐term glucocorticoid use, oxidative stress, and neuroendocrine dysregulation. However, the exact biological mechanisms underlying asthma‐related hippocampal volume changes require further investigation. Clinically, early identification and intervention for central nervous system complications in asthma, particularly in patients undergoing long‐term glucocorticoid treatment, may be critical for preserving neurocognitive health. In conclusion, this study not only provides genetic evidence for the causal relationship between asthma and hippocampal volume changes but also opens new avenues for research on the central nervous system complications of asthma. However, given that the findings are based primarily on European populations, future studies should extend these results to other ethnic groups and incorporate long‐term follow‐up data to further validate and expand these findings.

## Author Contributions

**Dandan Han**: conceptualization, data curation, formal analysis, investigation, methodology, software, validation, visualization, writing–original draft, writing–review and editing. **Shaohua Zhao**: conceptualization, funding acquisition, project administration, resources, supervision, validation, writing–review and editing.

### Peer Review

The peer review history for this article is available at https://publons.com/publon/10.1002/brb3.70560.

## Supporting information




**Supplementary Table 1** Detailed information on hippocampal volume imaging‐derived phenotypes (IDPs).
**Supplementary Table 2** Detailed information on genome‐wide association studies and datasets utilized in this study.
**Supplementary Table 3** Parameters and processing details of T1‐weighted structural imaging.
**Supplementary Table 4** Characteristics and *F*‐statistics of genetic variants associated with asthma.
**Supplementary Table 5** Effect estimates of the association between asthma and eight hippocampal volume IDPs risk in MR analysis.
**Supplementary Table 6** Heterogeneity and horizontal pleiotropy analyses for eight hippocampal volume IDPs in relation to asthma.
**Supplementary Table 7** Characteristics and *F*‐statistics of genetic variants associated with eight hippocampal volume IDPs.
**Supplementary Table 8** Effect estimates of the association between eight hippocampal volume IDPs and asthma risk in reverse MR analysis.
**Supplementary Figure 1** Causal effect of each SNP used as an instrumental variable on the outcome.
**Supplementary Figure 2** Key results of reverse Mendelian randomization analysis.

## Data Availability

The GWAS data used for asthma in this study can be obtained from the MRC IEU Open GWAS database (https://gwas.mrcieu.ac.uk/). The summary statistics for the GWAS results of all hippocampal volume IDPs can be accessed through the GWAS Catalog. For more detailed information about the GWAS of hippocampal volume IDPs, please refer to the Oxford Brain Imaging Genetics (BIG40) network server (https://open.win.ox.ac.uk/ukbiobank/big40/).
