# Supplementary material for: The Causal Relationship Between Asthma and Hippocampal Volume: A Study Based on Bidirectional Mendelian Randomization Analysis
Source: Brain Behav. 2025 May 26;15(5):e70560. doi: 10.1002/brb3.70560 (PMC12105112; doi:10.1002/brb3.70560)
Supplement: Supplementary file 1 — Supplementary Table 1 Detailed information on hippocampal volume imaging‐derived phenotypes (IDPs). Supplementary Table 2 Detailed information on genome‐wide association studies and datasets utilized in this study. Supplementary Table 3 Parameters and processing details of T1‐weighted structural imaging. Supplementary Table 4 Characteristics and F‐statistics of genetic variants associated with asthma. Supplementary Table 5 Effect estimates of the association between asthma and eight hippocampal volume IDPs risk in MR analysis. Supplementary Table 6 Heterogeneity and horizontal pleiotropy analyses for eight hippocampal volume IDPs in relation to asthma. Supplementary Table 7 Characteristics and F‐statistics of genetic variants associated with eight hippocampal volume IDPs. Supplementary Table 8 Effect estimates of the association between eight hippocampal volume IDPs and asthma risk in reverse MR analysis. Supplementary Figure 1 Causal effect of each SNP used as an instrumental variable on the outcome. Supplementary Figure 2 Key results of reverse Mendelian randomization analysis. [file BRB3-15-e70560-s001.docx]

**Supporting Information**

**Supplementary Table 1.** Detailed information on hippocampal volume imaging-derived phenotypes (IDPs).

| **IDP name** | **UK Biobank data-field ID** | **IDP unit** | **Measurement** | **Classification** | **Methods** | **IDP full description^1,2^** |
| --- | --- | --- | --- | --- | --- | --- |
| T1 image left hippocampus volume | 25019 | mm3 | regional and tissue volume | Structural MRI | T1-FIRST^3^ | Volume of left hippocampus (from T1 brain image) |
| T1 image right hippocampus volume | 25020 | mm3 | regional and tissue volume | Structural MRI | T1-FIRST | Volume of right hippocampus (from T1 brain image) |
| Left hippocampus grey matter volume | 25886 | mm3 | regional and tissue volume | Structural MRI | T1 FAST^4^ | Volume of grey matter in Left Hippocampus |
| Right hippocampus grey matter volume | 25887 | mm3 | regional and tissue volume | Structural MRI | T1 FAST | Volume of grey matter in Right Hippocampus |
| Left hippocampal volume from aseg | 26562 | mm3 | regional and tissue volume | Structural MRI | T1-weighted imaging with aseg | Volume of Hippocampus in the left hemisphere generated by subcortical volumetric segmentation (aseg) |
| Right hippocampal volume from aseg | 26593 | mm3 | regional and tissue volume | Structural MRI | T1-weighted imaging with aseg | Volume of Hippocampus in the right hemisphere generated by subcortical volumetric segmentation (aseg) |
| Left Whole-hippocampus volume from sub-seg | 26641 | mm3 | regional and tissue volume | Structural MRI | T1-weighted imaging with sub-segmentation | Volume of Whole-hippocampus in the left hemisphere generated by subcortical volumetric sub-segmentation of the Hippocampal Subfields |
| Right Whole-hippocampus volume from sub-seg | 26663 | mm3 | regional and tissue volume | Structural MRI | T1-weighted imaging with sub-segmentation | Volume of Whole-hippocampus in the right hemisphere generated by subcortical volumetric sub-segmentation of the Hippocampal Subfields |

Abbreviations: IDP, Imaging-derived phenotype; MRI, Magnetic resonance imaging; FIRST, FMRIB's integrated registration and segmentation tool; FAST, FMRIB's automated segmentation tool; aseg, Automated segmentation; sub-seg, Sub-segmentation.

**Supplementary Table 2.** Detailed information on genome-wide association studies and datasets utilized in this study.

| **Exposure or outcome** | **Population** | **Sample size** | **Number of SNPs** | **Author** | **Links for data** | **PMID** |
| --- | --- | --- | --- | --- | --- | --- |
| Asthma | European | 155386 | 16380170 | NA | https://gwas.mrcieu.ac.uk/datasets/finn-b-ASTHMA_MODE/ | NA |
| T1 image left hippocampus volume | European | 33211 | 17103079 | Elliott LT | https://gwas.mrcieu.ac.uk/datasets/ubm-b-19/ | 33875891 |
| T1 image right hippocampus volume | European | 33211 | 17103079 | Elliott LT | https://gwas.mrcieu.ac.uk/datasets/ubm-b-20/ | 33875891 |
| Left hippocampus grey matter volume | European | 33219 | 17103079 | Elliott LT | https://gwas.mrcieu.ac.uk/datasets/ubm-b-130/ | 33875891 |
| Right hippocampus grey matter volume | European | 33219 | 17103079 | Elliott LT | https://gwas.mrcieu.ac.uk/datasets/ubm-b-131/ | 33875891 |
| Left hippocampal volume from aseg | European | 31968 | 17103079 | Elliott LT | https://gwas.mrcieu.ac.uk/datasets/ubm-b-199/ | 33875891 |
| Right hippocampal volume from aseg | European | 31968 | 17103079 | Elliott LT | https://gwas.mrcieu.ac.uk/datasets/ubm-b-216/ | 33875891 |
| Left Whole-hippocampus volume from sub-seg | European | 31968 | 17103079 | Elliott LT | https://gwas.mrcieu.ac.uk/datasets/ubm-b-264/ | 33875891 |
| Right Whole-hippocampus volume from sub-seg | European | 31968 | 17103079 | Elliott LT | https://gwas.mrcieu.ac.uk/datasets/ubm-b-286/ | 33875891 |

**Supplementary Table 3.** Parameters and processing details of T1-weighted structural imaging.

| **Type of MRI^1,2^** | **Imaging hardware** | **Setup** | **Image processing** |
| --- | --- | --- | --- |
| A T1-weighted structural image. T1-weighted imaging is a structural technique with high-resolution depiction of brain anatomy, having strong contrast between grey and white matter, reflecting differences in the interaction of water with surrounding tissues (tissue T1 relaxation times). This modality provides IDPs primarily relating to volumes of brain tissues and structures. It is also critical for calculations of cross-subject and cross-modality alignments, needed in order to process all other brain modalities. | The scanner is a standard Siemens Skyra 3T running VD13A SP4 (as of October 2015), with a standard Siemens 32-channel RF receive head coil.  Initial data was from a single scanner dedicated to UK Biobank imaging, in Cheadle Manchester. In 2017 two further identical centres (in Newcastle and Reading) began scanning. | It is critical to achieve maximally consistent spatial coverage of scans in the presence of diﬀerences in subject positioning and head size. For each scan, the ﬁeld-of-view is automatically determined based on Siemens’ auto-align software, which aligns a scout scan to an atlas. In the infrequent situation where auto-align failed, alignment was set by the radiographer. T1 structural is acquired using straight sagittal orientation (i.e., with the field-of-view aligned to the scanner axes) . | Resolution: 1x1x1 mm  Field-of-view: 208x256x256 matrix  Duration: 5 minutes  3D MPRAGE, sagittal, in-plane acceleration iPAT=2, prescan-normalise  The superior-inferior field-of-view is large (256mm), at little cost, in order to include reasonable amounts of neck/mouth, as those  areas will be of interest to some researchers. |

**Supplementary Table 4.** Characteristics and *F*-statistics of genetic variants associated with asthma.

| **Number** | **SNP** | **Chr** | **Position** | **Effect allele** | **Beta** | **SE** | ***P*-value** | ***F*-statistic** |
| --- | --- | --- | --- | --- | --- | --- | --- | --- |
| 1 | rs11678975 | 2 | 103043739 | A | 0.095 | 0.013 | 3.00E-13 | 53.515 |
| 2 | rs62192043 | 2 | 242711282 | A | -0.107 | 0.014 | 4.63E-14 | 56.992 |
| 3 | rs72837868 | 2 | 112010094 | G | 0.120 | 0.019 | 7.21E-11 | 42.355 |
| 4 | rs1837253 | 5 | 110401872 | C | 0.137 | 0.014 | 1.15E-22 | 95.760 |
| 5 | rs6894249 | 5 | 131797547 | G | 0.091 | 0.012 | 1.07E-14 | 59.866 |
| 6 | rs1010473 | 6 | 90856878 | T | -0.081 | 0.014 | 2.87E-09 | 35.129 |
| 7 | rs35242582 | 6 | 32600057 | G | -0.129 | 0.015 | 8.59E-19 | 78.553 |
| 8 | rs4713555 | 6 | 32575524 | T | -0.079 | 0.013 | 3.71E-09 | 34.581 |
| 9 | rs11137222 | 9 | 140672257 | T | 0.175 | 0.032 | 4.83E-08 | 29.737 |
| 10 | rs7035413 | 9 | 6243119 | G | 0.131 | 0.015 | 3.42E-18 | 75.725 |
| 11 | rs827631 | 10 | 9015230 | A | 0.095 | 0.015 | 2.44E-10 | 40.027 |
| 12 | rs7126418 | 11 | 76292573 | T | 0.071 | 0.012 | 3.50E-09 | 34.810 |
| 13 | rs9517711 | 13 | 100074280 | A | 0.066 | 0.012 | 2.49E-08 | 30.854 |
| 14 | rs17293632 | 15 | 67442596 | T | 0.098 | 0.013 | 2.02E-13 | 53.705 |
| 15 | rs74630264 | 16 | 27316975 | A | -0.159 | 0.022 | 3.46E-13 | 53.063 |
| 16 | rs8074437 | 17 | 38076137 | G | 0.101 | 0.012 | 2.86E-17 | 71.324 |
| 17 | rs72699 | 17 | 47328890 | C | -0.065 | 0.012 | 4.65E-08 | 29.971 |
| 18 | rs118013485 | 19 | 33726577 | A | -0.111 | 0.020 | 3.41E-08 | 30.387 |
| 19 | rs11667612 | 19 | 11810494 | T | 0.150 | 0.026 | 1.25E-08 | 32.455 |

Abbreviations: Chr, Chromosome; SE, Standard error; SNP, Single nucleotide polymorphism.

**Supplementary Table 5.** Effect estimates of the association between asthma and eight hippocampal volume IDPs risk in MR analysis.

| **Outcome** | **Method** | **Number of SNPs** | **Beta** | **SE** | **OR** | **or_lci95** | **or_uci95** | P-value | ***P*_FDR_** |
| --- | --- | --- | --- | --- | --- | --- | --- | --- | --- |
| T1 image left hippocampus volume | MR Egger | 16 | -0.024 | 0.105 | 0.976 | 0.794 | 1.199 | 0.820 | 0.220 |
|  | Weighted median | 16 | -0.069 | 0.032 | 0.933 | 0.877 | 0.993 | 0.029 | 0.037 |
|  | Inverse variance weighted | 16 | -0.065 | 0.024 | 0.937 | 0.894 | 0.981 | 0.006 | 0.013 |
|  | Simple mode | 16 | -0.081 | 0.045 | 0.922 | 0.844 | 1.007 | 0.090 | 0.062 |
|  | Weighted mode | 16 | -0.072 | 0.040 | 0.930 | 0.860 | 1.006 | 0.091 | 0.062 |
| T1 image right hippocampus volume | MR Egger | 16 | -0.074 | 0.105 | 0.929 | 0.756 | 1.142 | 0.495 | 0.146 |
|  | Weighted median | 16 | -0.087 | 0.032 | 0.917 | 0.861 | 0.976 | 0.006 | 0.013 |
|  | Inverse variance weighted | 16 | -0.068 | 0.024 | 0.934 | 0.892 | 0.978 | 0.004 | 0.013 |
|  | Simple mode | 16 | -0.085 | 0.053 | 0.919 | 0.828 | 1.020 | 0.131 | 0.072 |
|  | Weighted mode | 16 | -0.102 | 0.050 | 0.903 | 0.820 | 0.996 | 0.059 | 0.052 |
| Left hippocampus grey matter volume | MR Egger | 16 | -0.168 | 0.122 | 0.846 | 0.665 | 1.075 | 0.192 | 0.082 |
|  | Weighted median | 16 | -0.036 | 0.034 | 0.965 | 0.903 | 1.031 | 0.294 | 0.104 |
|  | Inverse variance weighted | 16 | -0.034 | 0.028 | 0.966 | 0.915 | 1.020 | 0.216 | 0.085 |
|  | Simple mode | 16 | -0.050 | 0.048 | 0.951 | 0.866 | 1.045 | 0.314 | 0.108 |
|  | Weighted mode | 16 | -0.056 | 0.042 | 0.946 | 0.871 | 1.027 | 0.206 | 0.084 |
| Right hippocampus grey matter volume | MR Egger | 16 | -0.110 | 0.122 | 0.895 | 0.704 | 1.138 | 0.382 | 0.122 |
|  | Weighted median | 16 | -0.061 | 0.034 | 0.941 | 0.881 | 1.006 | 0.072 | 0.057 |
|  | Inverse variance weighted | 16 | -0.081 | 0.027 | 0.922 | 0.875 | 0.972 | 0.002 | 0.013 |
|  | Simple mode | 16 | -0.055 | 0.059 | 0.947 | 0.844 | 1.062 | 0.366 | 0.119 |
|  | Weighted mode | 16 | -0.058 | 0.050 | 0.944 | 0.856 | 1.041 | 0.267 | 0.098 |
| Left hippocampal volume from aseg | MR Egger | 16 | -0.052 | 0.107 | 0.949 | 0.769 | 1.171 | 0.635 | 0.179 |
|  | Weighted median | 16 | -0.043 | 0.032 | 0.958 | 0.899 | 1.021 | 0.186 | 0.081 |
|  | Inverse variance weighted | 16 | -0.054 | 0.024 | 0.947 | 0.903 | 0.993 | 0.024 | 0.034 |
|  | Simple mode | 16 | 0.001 | 0.056 | 1.001 | 0.897 | 1.117 | 0.988 | 0.254 |
|  | Weighted mode | 16 | -0.033 | 0.045 | 0.967 | 0.886 | 1.056 | 0.470 | 0.141 |
| Right hippocampal volume from aseg | MR Egger | 16 | -0.089 | 0.107 | 0.915 | 0.741 | 1.129 | 0.420 | 0.130 |
|  | Weighted median | 16 | -0.069 | 0.033 | 0.933 | 0.874 | 0.997 | 0.039 | 0.043 |
|  | Inverse variance weighted | 16 | -0.058 | 0.024 | 0.944 | 0.900 | 0.989 | 0.016 | 0.026 |
|  | Simple mode | 16 | -0.051 | 0.049 | 0.951 | 0.863 | 1.047 | 0.318 | 0.109 |
|  | Weighted mode | 16 | -0.069 | 0.046 | 0.933 | 0.852 | 1.021 | 0.154 | 0.077 |
| Left Whole-hippocampus volume from sub-seg | MR Egger | 16 | -0.035 | 0.112 | 0.965 | 0.775 | 1.203 | 0.759 | 0.207 |
|  | Weighted median | 16 | -0.050 | 0.034 | 0.952 | 0.891 | 1.017 | 0.141 | 0.074 |
|  | Inverse variance weighted | 16 | -0.045 | 0.024 | 0.956 | 0.911 | 1.003 | 0.063 | 0.054 |
|  | Simple mode | 16 | -0.088 | 0.053 | 0.916 | 0.825 | 1.016 | 0.119 | 0.070 |
|  | Weighted mode | 16 | -0.062 | 0.045 | 0.940 | 0.861 | 1.027 | 0.189 | 0.082 |
| Right Whole-hippocampus volume from sub-seg | MR Egger | 16 | -0.044 | 0.107 | 0.957 | 0.776 | 1.181 | 0.687 | 0.191 |
|  | Weighted median | 16 | -0.064 | 0.032 | 0.938 | 0.881 | 0.999 | 0.045 | 0.046 |
|  | Inverse variance weighted | 16 | -0.069 | 0.024 | 0.933 | 0.890 | 0.979 | 0.004 | 0.013 |
|  | Simple mode | 16 | -0.074 | 0.054 | 0.929 | 0.836 | 1.032 | 0.190 | 0.082 |
|  | Weighted mode | 16 | -0.062 | 0.046 | 0.940 | 0.859 | 1.028 | 0.197 | 0.083 |

Abbreviations: SE, Standard error; SNP, Single nucleotide polymorphism; OR, Odds ratio; CI, Confidence interval.

**Supplementary Table 6.** Heterogeneity and horizontal pleiotropy analyses for eight hippocampal volume IDPs in relation to asthma.

| **Exposure** | **Outcome** | **Heterogeneity** | | | **Horizontal pleiotropy** | | | **MR-PRESSO *P*-value** |
| --- | --- | --- | --- | --- | --- | --- | --- | --- |
|  |  | **IVW Q** | **IVW Q df** | **IVW *P*** | **Egger intercept** | **SE** | ***P*** |  |
| Asthma | T1 image left hippocampus volume | 13.490 | 15 | 0.565 | -0.004 | 0.011 | 0.697 | 0.629 |
|  | T1 image right hippocampus volume | 13.136 | 15 | 0.592 | 0.001 | 0.011 | 0.959 | 0.600 |
|  | Left hippocampus grey matter volume | 20.676 | 15 | 0.147 | 0.014 | 0.013 | 0.282 | 0.169 |
|  | Right hippocampus grey matter volume | 19.074 | 15 | 0.210 | 0.003 | 0.013 | 0.810 | 0.226 |
|  | Left hippocampal volume from aseg | 13.349 | 15 | 0.575 | 0.000 | 0.011 | 0.983 | 0.624 |
|  | Right hippocampal volume from aseg | 9.816 | 15 | 0.831 | 0.003 | 0.011 | 0.770 | 0.845 |
|  | Left Whole-hippocampus volume from sub-seg | 15.346 | 15 | 0.427 | -0.001 | 0.012 | 0.927 | 0.454 |
|  | Right Whole-hippocampus volume from sub-seg | 13.021 | 15 | 0.601 | -0.003 | 0.011 | 0.815 | 0.604 |

Abbreviations: IVW, Inverse variance weighted; Q, Cochran’s Q test estimate; df, Cochran’s Q test degrees of freedom; SE, Standard error.


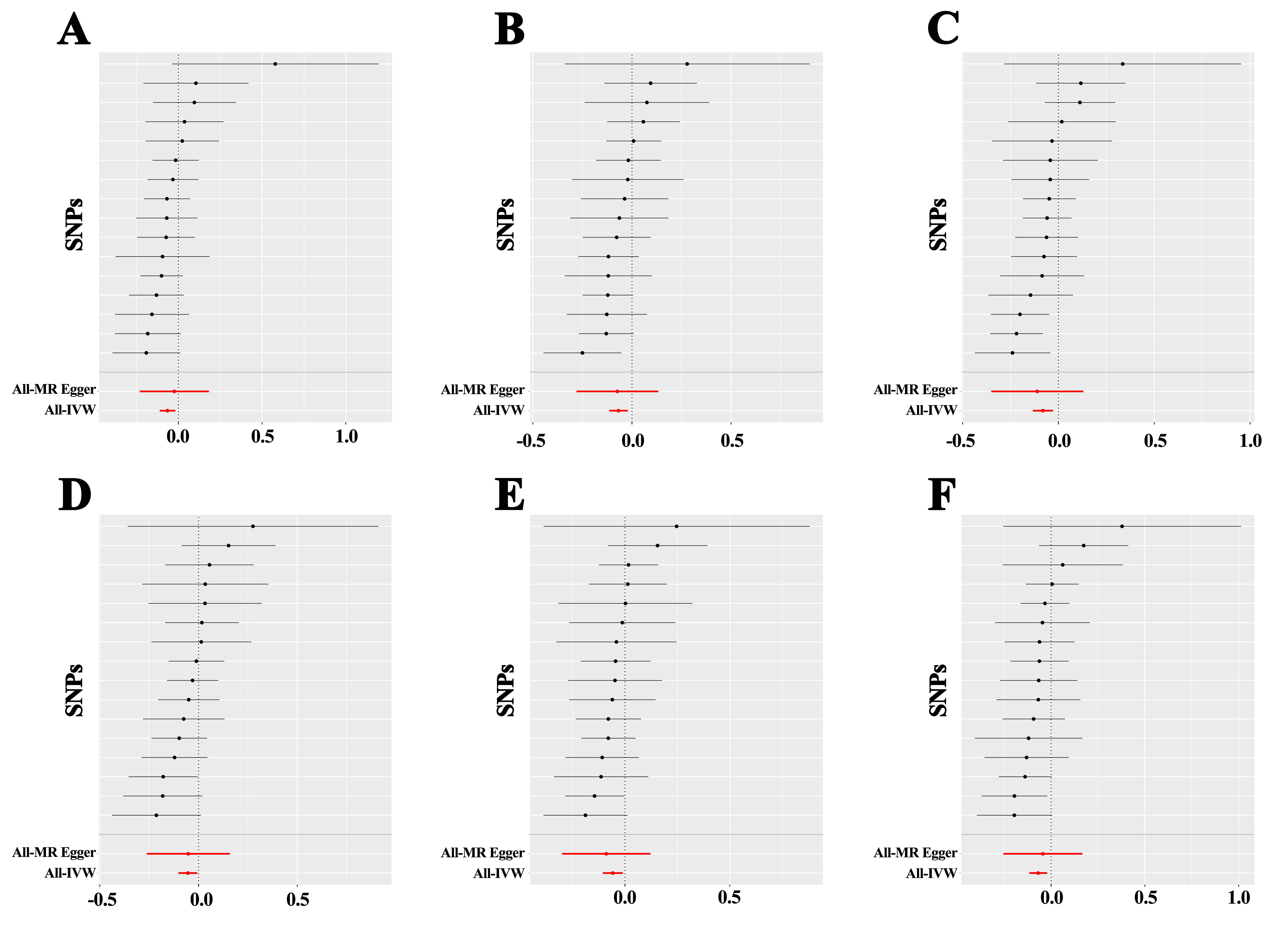


**Supplementary Figure 1.** Causal effect of each SNP used as an instrumental variable on the outcome. **(**A) Effect estimates of asthma on T1 image left hippocampus volume. (B) Effect estimates of asthma on T1 image right hippocampus volume. (C) Effect estimates of asthma on right hippocampus grey matter volume. (D) Effect estimates of asthma on left hippocampal volume from aseg. (E) Effect estimates of asthma on right hippocampal volume from aseg. (F) Effect estimates of asthma on right whole-hippocampus volume from sub-seg.

**Supplementary Table 7.** Characteristics and *F*-statistics of genetic variants associated with eight hippocampal volume IDPs.

| **Exposure** | **Number** | **SNP** | **Chr** | **Position** | **Effect allele** | **Beta** | **SE** | ***P*-value** | ***F*-statistic** |
| --- | --- | --- | --- | --- | --- | --- | --- | --- | --- |
| T1 image left hippocampus volume | 1 | rs17178006 | 12 | 65718299 | G | -0.099 | 0.012 | 1.57E-16 | 68.159 |
|  | 2 | rs2970932 | 2 | 162858200 | G | -0.050 | 0.008 | 2.34E-10 | 40.187 |
|  | 3 | rs3800230 | 6 | 108998128 | G | -0.067 | 0.012 | 1.95E-08 | 31.565 |
|  | 5 | rs4624605 | 3 | 190640121 | T | 0.045 | 0.008 | 3.50E-08 | 30.422 |
|  | 6 | rs6690450 | 1 | 54854079 | A | -0.053 | 0.008 | 6.52E-12 | 47.203 |
|  | 7 | rs7040792 | 9 | 119248993 | C | 0.060 | 0.008 | 8.85E-14 | 55.655 |
| T1 image right hippocampus volume | 1 | rs10888849 | 1 | 54844913 | A | -0.044 | 0.008 | 8.80E-09 | 33.106 |
|  | 2 | rs118125918 | 15 | 37201699 | G | 0.139 | 0.025 | 3.76E-08 | 30.282 |
|  | 3 | rs12523793 | 6 | 149973764 | A | 0.048 | 0.008 | 4.51E-09 | 34.410 |
|  | 5 | rs4624605 | 3 | 190640121 | T | 0.054 | 0.008 | 3.16E-11 | 44.109 |
|  | 6 | rs6432708 | 2 | 162891848 | T | -0.051 | 0.008 | 1.27E-10 | 41.374 |
|  | 8 | rs73123652 | 12 | 65874956 | C | -0.085 | 0.012 | 5.62E-12 | 47.494 |
|  | 9 | rs73220476 | 12 | 117287112 | T | 0.080 | 0.011 | 7.29E-13 | 51.505 |
| Left hippocampus grey matter volume | 1 | rs1052607 | 1 | 46499526 | G | -0.082 | 0.015 | 2.01E-08 | 31.497 |
|  | 2 | rs1075472 | 14 | 93108131 | G | 0.056 | 0.010 | 1.67E-08 | 31.861 |
|  | 3 | rs12111923 | 7 | 148885877 | A | -0.060 | 0.009 | 1.20E-11 | 45.997 |
|  | 4 | rs13176921 | 5 | 82860348 | G | 0.076 | 0.010 | 5.05E-15 | 61.302 |
|  | 5 | rs146607495 | 12 | 117319202 | T | 0.180 | 0.013 | 6.47E-42 | 184.516 |
|  | 6 | rs2015333 | 12 | 65862790 | C | 0.052 | 0.008 | 3.53E-11 | 43.887 |
|  | 8 | rs279888 | 9 | 978833 | A | -0.045 | 0.008 | 1.19E-08 | 32.520 |
|  | 10 | rs4624605 | 3 | 190640121 | T | -0.045 | 0.008 | 2.85E-08 | 30.822 |
|  | 11 | rs6432708 | 2 | 162891848 | T | -0.060 | 0.008 | 3.93E-14 | 57.254 |
|  | 12 | rs6690450 | 1 | 54854079 | A | -0.054 | 0.008 | 2.61E-12 | 48.999 |
|  | 13 | rs67744585 | 3 | 190670215 | G | -0.046 | 0.008 | 1.46E-08 | 32.119 |
|  | 14 | rs7030607 | 9 | 119245183 | A | 0.066 | 0.008 | 5.36E-16 | 65.724 |
|  | 15 | rs72761265 | 5 | 66145563 | G | 0.048 | 0.008 | 4.52E-09 | 34.404 |
|  | 16 | rs8029552 | 15 | 98442822 | C | -0.052 | 0.008 | 3.34E-11 | 43.998 |
| Right hippocampus grey matter volume | 1 | rs1052607 | 1 | 46499526 | G | -0.081 | 0.015 | 3.81E-08 | 30.260 |
|  | 2 | rs10784446 | 12 | 65764979 | G | -0.061 | 0.008 | 3.27E-14 | 57.615 |
|  | 3 | rs11865702 | 16 | 70681298 | G | 0.059 | 0.008 | 6.00E-14 | 56.419 |
|  | 4 | rs146607495 | 12 | 117319202 | T | 0.180 | 0.013 | 9.53E-42 | 183.759 |
|  | 5 | rs17214697 | 5 | 66118105 | G | 0.045 | 0.008 | 2.26E-08 | 31.273 |
|  | 6 | rs1962479 | 2 | 48179295 | A | 0.047 | 0.008 | 8.83E-09 | 33.098 |
|  | 8 | rs279888 | 9 | 978833 | A | -0.049 | 0.008 | 5.58E-10 | 38.486 |
|  | 9 | rs28758826 | 15 | 98443571 | G | -0.057 | 0.008 | 3.13E-13 | 53.166 |
|  | 11 | rs4862292 | 4 | 184958217 | C | -0.048 | 0.008 | 9.06E-10 | 37.539 |
|  | 12 | rs6432708 | 2 | 162891848 | T | -0.073 | 0.008 | 3.24E-20 | 84.943 |
|  | 13 | rs6690450 | 1 | 54854079 | A | -0.052 | 0.008 | 1.68E-11 | 45.345 |
|  | 14 | rs7030607 | 9 | 119245183 | A | 0.064 | 0.008 | 2.22E-15 | 62.922 |
| Left hippocampal volume from aseg | 2 | rs12817240 | 12 | 41846898 | C | -0.082 | 0.015 | 2.91E-08 | 30.779 |
|  | 3 | rs146607495 | 12 | 117319202 | T | 0.181 | 0.014 | 6.61E-41 | 179.894 |
|  | 4 | rs17178006 | 12 | 65718299 | G | -0.137 | 0.012 | 2.74E-29 | 126.470 |
|  | 5 | rs2578475 | 12 | 4007324 | G | -0.049 | 0.008 | 4.94E-09 | 34.232 |
|  | 6 | rs28758826 | 15 | 98443571 | G | -0.047 | 0.008 | 3.82E-09 | 34.732 |
|  | 7 | rs2970931 | 2 | 162873188 | C | -0.071 | 0.008 | 9.08E-19 | 78.349 |
|  | 9 | rs61192764 | 6 | 108995187 | G | -0.074 | 0.012 | 1.22E-09 | 36.955 |
|  | 10 | rs61785580 | 1 | 46402849 | T | -0.099 | 0.015 | 1.47E-10 | 41.087 |
|  | 11 | rs6699195 | 1 | 54841838 | C | -0.065 | 0.008 | 1.26E-16 | 68.591 |
|  | 12 | rs7030607 | 9 | 119245183 | A | 0.079 | 0.008 | 1.56E-21 | 90.961 |
| Right hippocampal volume from aseg | 1 | rs146607495 | 12 | 117319202 | T | 0.169 | 0.014 | 7.23E-36 | 156.703 |
|  | 2 | rs17178006 | 12 | 65718299 | G | -0.146 | 0.012 | 3.44E-33 | 144.394 |
|  | 3 | rs2559508 | 10 | 126552361 | A | -0.066 | 0.009 | 3.49E-14 | 57.490 |
|  | 4 | rs28758826 | 15 | 98443571 | G | -0.052 | 0.008 | 8.05E-11 | 42.273 |
|  | 5 | rs33931638 | 1 | 46500251 | A | -0.084 | 0.015 | 2.53E-08 | 31.055 |
|  | 6 | rs3924855 | 16 | 29954955 | G | -0.046 | 0.008 | 9.10E-09 | 33.042 |
|  | 7 | rs4038372 | 6 | 150045578 | T | 0.061 | 0.008 | 2.32E-13 | 53.757 |
|  | 8 | rs62449208 | 7 | 31446101 | G | -0.063 | 0.011 | 3.27E-08 | 30.554 |
|  | 9 | rs6432708 | 2 | 162891848 | T | -0.078 | 0.008 | 4.48E-22 | 93.446 |
|  | 10 | rs6690450 | 1 | 54854079 | A | -0.059 | 0.008 | 5.66E-14 | 56.534 |
|  | 11 | rs7030607 | 9 | 119245183 | A | 0.073 | 0.008 | 1.25E-18 | 77.710 |
|  | 12 | rs7752159 | 6 | 111787820 | C | -0.057 | 0.009 | 1.35E-09 | 36.757 |
| Left Whole-hippocampus volume from sub-seg | 1 | rs1055256 | 10 | 126446592 | G | -0.073 | 0.008 | 4.47E-20 | 84.311 |
|  | 2 | rs12423463 | 12 | 41903274 | A | -0.082 | 0.015 | 3.25E-08 | 30.566 |
|  | 3 | rs146607495 | 12 | 117319202 | T | 0.179 | 0.014 | 7.40E-40 | 175.060 |
|  | 4 | rs17178006 | 12 | 65718299 | G | -0.137 | 0.012 | 3.44E-29 | 126.028 |
|  | 6 | rs213490 | 1 | 54854984 | A | -0.068 | 0.008 | 6.97E-18 | 74.316 |
|  | 7 | rs2909456 | 2 | 162836954 | T | -0.083 | 0.008 | 4.55E-25 | 107.139 |
|  | 8 | rs3809627 | 16 | 30103160 | A | 0.045 | 0.008 | 3.08E-08 | 30.672 |
|  | 10 | rs4947122 | 6 | 111870090 | T | -0.058 | 0.010 | 3.66E-09 | 34.816 |
|  | 11 | rs61785580 | 1 | 46402849 | T | -0.092 | 0.015 | 1.97E-09 | 36.022 |
|  | 12 | rs7030607 | 9 | 119245183 | A | 0.078 | 0.008 | 3.70E-21 | 89.257 |
|  | 13 | rs7221167 | 17 | 43933307 | C | -0.048 | 0.008 | 2.31E-09 | 35.715 |
|  | 14 | rs72761269 | 5 | 66153524 | A | 0.048 | 0.008 | 4.72E-09 | 34.322 |
|  | 15 | rs77870968 | 3 | 44035493 | G | 0.129 | 0.023 | 2.83E-08 | 30.834 |
|  | 16 | rs9783701 | 15 | 98433406 | T | -0.052 | 0.008 | 2.70E-10 | 39.908 |
| Right Whole-hippocampus volume from sub-seg | 1 | rs10888849 | 1 | 54844913 | A | -0.070 | 0.008 | 7.78E-19 | 78.655 |
|  | 2 | rs11733245 | 4 | 184958962 | G | -0.046 | 0.008 | 1.64E-08 | 31.899 |
|  | 4 | rs146607495 | 12 | 117319202 | T | 0.182 | 0.014 | 2.12E-41 | 182.170 |
|  | 5 | rs17178006 | 12 | 65718299 | G | -0.151 | 0.012 | 2.31E-35 | 154.398 |
|  | 6 | rs28753977 | 7 | 155807118 | C | 0.053 | 0.009 | 1.06E-08 | 32.752 |
|  | 7 | rs33931638 | 1 | 46500251 | A | -0.089 | 0.015 | 3.48E-09 | 34.915 |
|  | 8 | rs3809627 | 16 | 30103160 | A | 0.049 | 0.008 | 1.88E-09 | 36.116 |
|  | 10 | rs4038372 | 6 | 150045578 | T | 0.065 | 0.008 | 7.24E-15 | 60.589 |
|  | 12 | rs62449208 | 7 | 31446101 | G | -0.064 | 0.011 | 1.94E-08 | 31.569 |
|  | 13 | rs6432708 | 2 | 162891848 | T | -0.082 | 0.008 | 2.65E-24 | 103.629 |
|  | 14 | rs7030607 | 9 | 119245183 | A | 0.081 | 0.008 | 1.13E-22 | 96.183 |
|  | 16 | rs8026749 | 15 | 37217621 | A | -0.047 | 0.008 | 1.85E-08 | 31.665 |
|  | 17 | rs8034069 | 15 | 98429818 | C | -0.067 | 0.009 | 8.30E-13 | 51.251 |
|  | 19 | rs9911432 | 17 | 7962160 | G | -0.044 | 0.008 | 4.57E-08 | 29.904 |

Abbreviations: Chr, Chromosome;SE, Standard error; SNP, Single nucleotide polymorphism.

**Supplementary Table 8.** Effect estimates of the association between eight hippocampal volume IDPs and asthma risk in reverse MR analysis.

| **Exposure** | **Method** | **Number of SNPs** | **Beta** | **SE** | **OR** | **or_lci95** | **or_uci95** | P-value | ***P*_FDR_** |
| --- | --- | --- | --- | --- | --- | --- | --- | --- | --- |
| T1 image left hippocampus volume | MR Egger | 6 | -0.777 | 0.564 | 0.460 | 0.152 | 1.390 | 0.241 | 1.000 |
|  | Weighted median | 6 | -0.146 | 0.127 | 0.864 | 0.673 | 1.109 | 0.250 | 1.000 |
|  | Inverse variance weighted | 6 | -0.198 | 0.118 | 0.821 | 0.651 | 1.035 | 0.095 | 1.000 |
|  | Simple mode | 6 | -0.147 | 0.185 | 0.863 | 0.601 | 1.240 | 0.463 | 1.000 |
|  | Weighted mode | 6 | -0.129 | 0.173 | 0.879 | 0.626 | 1.234 | 0.489 | 1.000 |
| T1 image right hippocampus volume | MR Egger | 7 | -0.247 | 0.485 | 0.781 | 0.302 | 2.020 | 0.632 | 1.000 |
|  | Weighted median | 7 | -0.021 | 0.123 | 0.979 | 0.769 | 1.246 | 0.862 | 1.000 |
|  | Inverse variance weighted | 7 | 0.035 | 0.138 | 1.036 | 0.791 | 1.357 | 0.797 | 1.000 |
|  | Simple mode | 7 | -0.094 | 0.178 | 0.910 | 0.642 | 1.290 | 0.616 | 1.000 |
|  | Weighted mode | 7 | -0.054 | 0.180 | 0.948 | 0.666 | 1.349 | 0.776 | 1.000 |
| Left hippocampus grey matter volume | MR Egger | 14 | 0.038 | 0.222 | 1.039 | 0.673 | 1.606 | 0.866 | 1.000 |
|  | Weighted median | 14 | -0.085 | 0.083 | 0.919 | 0.781 | 1.080 | 0.305 | 1.000 |
|  | Inverse variance weighted | 14 | -0.108 | 0.077 | 0.898 | 0.772 | 1.043 | 0.160 | 1.000 |
|  | Simple mode | 14 | -0.190 | 0.144 | 0.827 | 0.624 | 1.097 | 0.211 | 1.000 |
|  | Weighted mode | 14 | -0.051 | 0.108 | 0.950 | 0.769 | 1.174 | 0.645 | 1.000 |
| Right hippocampus grey matter volume | MR Egger | 12 | -0.051 | 0.244 | 0.951 | 0.590 | 1.533 | 0.839 | 1.000 |
|  | Weighted median | 12 | -0.016 | 0.085 | 0.984 | 0.833 | 1.162 | 0.847 | 1.000 |
|  | Inverse variance weighted | 12 | -0.054 | 0.082 | 0.948 | 0.807 | 1.113 | 0.514 | 1.000 |
|  | Simple mode | 12 | 0.023 | 0.170 | 1.024 | 0.734 | 1.428 | 0.893 | 1.000 |
|  | Weighted mode | 12 | 0.027 | 0.123 | 1.028 | 0.807 | 1.308 | 0.829 | 1.000 |
| Left hippocampal volume from aseg | MR Egger | 10 | 0.000 | 0.186 | 1.000 | 0.695 | 1.440 | 0.999 | 1.000 |
|  | Weighted median | 10 | -0.079 | 0.082 | 0.924 | 0.787 | 1.086 | 0.338 | 1.000 |
|  | Inverse variance weighted | 10 | -0.144 | 0.068 | 0.866 | 0.758 | 0.990 | 0.035 | 1.000 |
|  | Simple mode | 10 | -0.076 | 0.138 | 0.927 | 0.708 | 1.213 | 0.593 | 1.000 |
|  | Weighted mode | 10 | -0.038 | 0.110 | 0.963 | 0.777 | 1.193 | 0.735 | 1.000 |
| Right hippocampal volume from aseg | MR Egger | 12 | 0.119 | 0.275 | 1.126 | 0.657 | 1.930 | 0.674 | 1.000 |
|  | Weighted median | 12 | -0.064 | 0.081 | 0.938 | 0.800 | 1.099 | 0.429 | 1.000 |
|  | Inverse variance weighted | 12 | -0.096 | 0.094 | 0.908 | 0.756 | 1.092 | 0.306 | 1.000 |
|  | Simple mode | 12 | -0.061 | 0.119 | 0.941 | 0.744 | 1.189 | 0.619 | 1.000 |
|  | Weighted mode | 12 | -0.051 | 0.102 | 0.950 | 0.777 | 1.161 | 0.625 | 1.000 |
| Left Whole-hippocampus volume from sub-seg | MR Egger | 14 | -0.011 | 0.249 | 0.989 | 0.607 | 1.612 | 0.965 | 1.000 |
|  | Weighted median | 14 | -0.055 | 0.075 | 0.947 | 0.817 | 1.097 | 0.466 | 1.000 |
|  | Inverse variance weighted | 14 | -0.059 | 0.087 | 0.943 | 0.795 | 1.118 | 0.499 | 1.000 |
|  | Simple mode | 14 | -0.152 | 0.112 | 0.859 | 0.690 | 1.069 | 0.197 | 1.000 |
|  | Weighted mode | 14 | -0.061 | 0.087 | 0.941 | 0.794 | 1.115 | 0.492 | 1.000 |
| Right Whole-hippocampus volume from sub-seg | MR Egger | 14 | -0.039 | 0.218 | 0.962 | 0.627 | 1.475 | 0.862 | 1.000 |
|  | Weighted median | 14 | -0.042 | 0.073 | 0.959 | 0.830 | 1.107 | 0.565 | 1.000 |
|  | Inverse variance weighted | 14 | -0.042 | 0.080 | 0.959 | 0.821 | 1.121 | 0.602 | 1.000 |
|  | Simple mode | 14 | 0.077 | 0.109 | 1.080 | 0.872 | 1.338 | 0.491 | 1.000 |
|  | Weighted mode | 14 | 0.015 | 0.099 | 1.015 | 0.837 | 1.232 | 0.880 | 1.000 |

Abbreviations: SE, Standard error; SNP, Single nucleotide polymorphism; OR, Odds ratio; CI, Confidence interval.





**Supplementary Figure 2.** Key results of reverse Mendelian randomization analysis.

**References**

1 Elliott, L. T. *et al.* Genome-wide association studies of brain imaging phenotypes in UK Biobank. *Nature* **562**, 210-216, doi:10.1038/s41586-018-0571-7 (2018).

2 Smith, S. M. *et al.* An expanded set of genome-wide association studies of brain imaging phenotypes in UK Biobank. *Nat Neurosci* **24**, 737-745, doi:10.1038/s41593-021-00826-4 (2021).

3 Patenaude, B., Smith, S. M., Kennedy, D. N. & Jenkinson, M. A Bayesian model of shape and appearance for subcortical brain segmentation. *Neuroimage* **56**, 907-922, doi:10.1016/j.neuroimage.2011.02.046 (2011).

4 Zhang, Y., Brady, M. & Smith, S. Segmentation of brain MR images through a hidden Markov random field model and the expectation-maximization algorithm. *IEEE Trans Med Imaging* **20**, 45-57 (2001).
